# Supplementary material for: Clinical Evidence of Bee Venom Acupuncture for Ankle Pain: A Review of Clinical Research
Source: Toxins (Basel). 2025 May 21;17(5):257. doi: 10.3390/toxins17050257 (PMC12115534; doi:10.3390/toxins17050257)
Supplement: Supplementary file 1 [file toxins-17-00257-s001.zip › Supplementary file S1. List of existing literature on bee venom acupuncture.pdf]

Supplementary file S1. List of existing literature on bee venom acupuncture

Author(s)	Study Title	Research Method	Key Findings
**Jeong et al. (2025) [18]**	*Effectiveness of Bee Venom Injection for Parkinson's Disease: A Systematic Review*	Systematic review of clinical studies	BVA may improve Parkinson's disease symptoms with mild adverse effects; no severe reactions reported.
**Jeong et al. (2024) [19]**	*Bee Venom Acupuncture for Shoulder Pain: A Literature Review of Clinical Studies*	Literature review of clinical studies	BVA showed positive effects on shoulder pain, including post-stroke pain and rotator cuff syndrome.
**Kim et al. (2024) [20]**	*Clinical Effectiveness of Bee Venom Acupuncture for Bone Fractures and Potential Mechanisms: A Narrative Overview*	Narrative review	BVA may aid in pain relief and recovery from bone fractures; further rigorous trials needed.
**Sung & Lee et al. (2023) [21]**	*Bee Venom Acupuncture for Neck Pain: A Review of the Korean Literature*	Review of Korean clinical studies	BVA has been utilized in Korean clinical practice to alleviate neck pain, particularly in cases involving cervical herniated intervertebral discs
**Sung et al. (2022) [22]**	*Clinical Studies of Bee Venom Acupuncture for Lower Back Pain in the Korean Literature*	Review of Korean clinical studies	Bee venom acupuncture has been utilized in Korean clinical practice to alleviate lower back pain, particularly in cases involving lumbar disc herniation.
**Carpena et al. (2020) [23]**	*Bee Venom: An Updating Review of Its Bioactive Molecules and Its Health Applications*	Comprehensive literature review	Bee venom exhibits potential therapeutic effects in treating inflammatory, neurodegenerative, and oncological conditions, primarily due to its bioactive components such as melittin and phospholipase A2.
**Lin et al. (2020) [24]**	*Clinical Applications of Bee Venom Acupoint Injection*	Review of clinical applications	Bee venom acupoint injection has been applied in clinical settings to treat various conditions, including Parkinson's disease, neuropathic

			pain, Alzheimer's disease, intervertebral disc disease, spinal cord injury, musculoskeletal pain, arthritis, multiple sclerosis, skin diseases, and cancer, owing to its anti-inflammatory, anti-apoptotic, and analgesic properties.
**Zhang et al. (2018) [25]**	*Bee Venom Therapy: Potential Mechanisms and Therapeutic Applications*	Review of mechanisms and applications	Bee venom therapy demonstrates potential therapeutic effects in treating inflammatory conditions, neurodegenerative diseases, and certain cancers, attributed to its bioactive components like melittin and phospholipase A_2.
**Lee et al. (2014) [26]**	*Bee Venom Acupuncture for Rheumatoid Arthritis: A Systematic Review of Randomised Clinical Trials*	Systematic review of RCTs	BVA may reduce pain and improve quality of life in rheumatoid arthritis patients.
**Park et al. (2007) [27]**	*Therapeutic Application of Anti-Arthritis, Pain-Releasing, and Anti-Cancer Effects of Bee Venom and Its Constituent Compounds*	Review of therapeutic applications	Bee venom therapy has potential benefits for arthritis, pain relief, and cancer treatment.
