# Supplementary material for: Clinical Evidence of Bee Venom Acupuncture for Ankle Pain: A Review of Clinical Research
Source: Toxins (Basel). 2025 May 21;17(5):257. doi: 10.3390/toxins17050257 (PMC12115534; doi:10.3390/toxins17050257)
Supplement: Supplementary file 1 [file toxins-17-00257-s001.zip › Supplementary file S2. Characteristics of the bee venom used in the included clinical studies.pdf]

Supplementary file S2. Characteristics of the bee venom used in the included clinical studies

First author (year)	species of bee	Composition of bee venom	Production of bee venom
Ahn [28] (2002)<br>Ryu [29] (2003)<br>Lee [30] (2004)<br>Song [31] (2005)<br>Kim [33] (2006)	Apis mellifera	n.r.	Self-produced [1)]
Choi [32] (2006)<br>Seo [34] (2006)<br>Kang [36] (2008)	Apis mellifera	Melittin (main component 52%), apamin, adolapin, phospholipase A_2, hyaluronidase, histamine, dopamine and protease inhibitor	Prepared in the AJ Herbal Dispensary[2)]<br>(https://www.pharmacopuncture.co.kr/shop/index.html)
Choi [35] (2008)	n.r.	n.r.	n.r.
Park [37] (2011)<br>Won [38] (2014)	Apis mellifera	Melittin	Prepared in the AJ Herbal Dispensary[2)]<br>(https://www.pharmacopuncture.co.kr/shop/index.html)
Oh [39] (2015)<br>Kim [40] (2016)<br>Oh [41] (2017)	Apis mellifera	Melittin (main component 52%), apamin, adolapin, phospholipase A_2, hyaluronidase, histamine, dopamine and protease inhibitor	Prepared in the Jaseng Herbal Dispensary[2)]<br>(https://herb.jaseng.co.kr/)

* n.r.: not reported in study

[1)] Self-produced: Bee venom collected by electrically stimulating bees is processed, dried, and then diluted with normal saline

[2)] Prepared in the Herbal Dispensary: Bee venom dried powder registered with the Korea Food and Drug Administration (FDA) is used in a preparation process involving sterilization, dilution with normal saline, filling, and sealing. Detailed preparation procedures and related photographs are provided in a previous study [42].
